# Hallmarks of Skin Aging: Update

**DOI:** 10.14336/AD.2023.0321

**Published:** 2023-12-01

**Authors:** Shifeng Jin, Kezhu Li, Xuanru Zong, Seokchan Eun, Naoki Morimoto, Shu Guo

**Affiliations:** ^1^Department of Plastic Surgery, the First Hospital of China Medical University, Liaoning, China.; ^2^College of Animal Science and Veterinary Medicine, Shandong Agricultural University, Shandong, China.; ^3^Department of Plastic Surgery, Seoul National University College of Medicine, Seoul National University Bundang Hospital, Seongnam 463-707, Korea.; ^4^Department of Plastic Reconstructive Surgery, Graduate School of Medicine, Kyoto University, Kyoto, Japan.

**Keywords:** hallmarks, skin, aging

## Abstract

Aging is defined as impaired physiological integrity, decreased function, increased susceptibility to external risk factors and various diseases. Skin, the largest organ in our body, may become more vulnerable to insult as time goes by and behave as aged skin. Here, we systemically reviewed three categories including seven hallmarks of skin aging. These hallmarks including genomic instability and telomere attrition, epigenetic alterations and loss of proteostasis, deregulated nutrient-sensing, mitochondrial damage and dysfunction, cellular senescence, stem cell exhaustion/dysregulation, and altered intercellular communication. These seven hallmarks can generally be divided into three categories including (i) causes of damages as primary hallmarks in skin aging; (ii) responses to damage as antagonistic hallmarks in skin aging; and (iii) culprits of the phenotype as integrative hallmarks in skin aging.

## 1. Introduction

**Figure 1.**
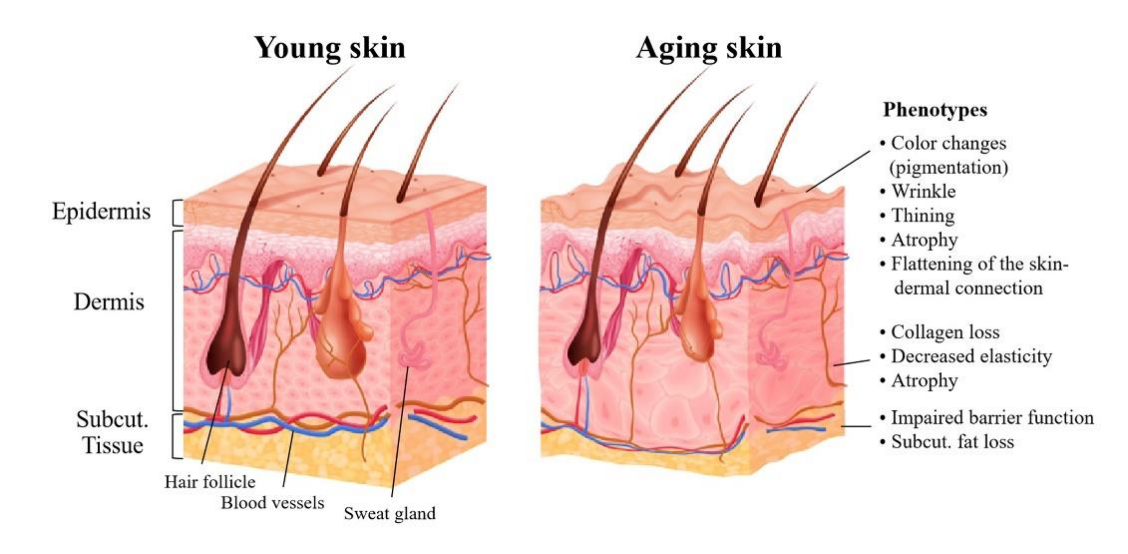
Skin structure and aging phenotypes.

Skin is the largest organ in the human body and has many complex functions. Skin is derived from embryonic origin of different cell types in the dermal layer and is divided into three layers: epidermis, dermis, and subcutaneous tissue. During development, the epidermis and dermis develop from the ectoderm and mesoderm, respectively. The epidermis develops from the ectoderm, which is an unspecified layer of progenitor cells covering the embryo after nerve formation, called the epidermal basal layer. The epidermal basal layer is rich in epidermal stem cell (ESC). The dermis mainly comes from the mesoderm under the ectoderm. The mesoderm is the main source of mesenchymal stem cells, which produces collagen fibroblasts (a component of blood vessels that feed the skin), subcutaneous fat cells and immune cells in the skin. In general, skin aging is characterized by color change (uneven pigmentation) and decreased elasticity, even skin atrophy, loss of underlying tissues and impaired barrier function [1]. It is characterized by phenotypic changes such as thinning and atrophy of the dermis and epidermis, subcutaneous fat loss, flattening of the skin-dermal connection, collagen loss, and disruption of the elastic fiber network (Fig. 1). These changes can ultimately destroy the structural integrity and function of different skin regions, leading to poor visible features and reduced elasticity, leaving aging skin vulnerable to injury and disease. With regard to skin aging, it is considered to be the superposition of benign skin phenotypes, indicating that histological and morphological changes are continuous and inevitable, which are caused by internal and external factors, with genetic and temporal influences constituting the former and environmental influences constituting the latter [2].

**Figure 2.**
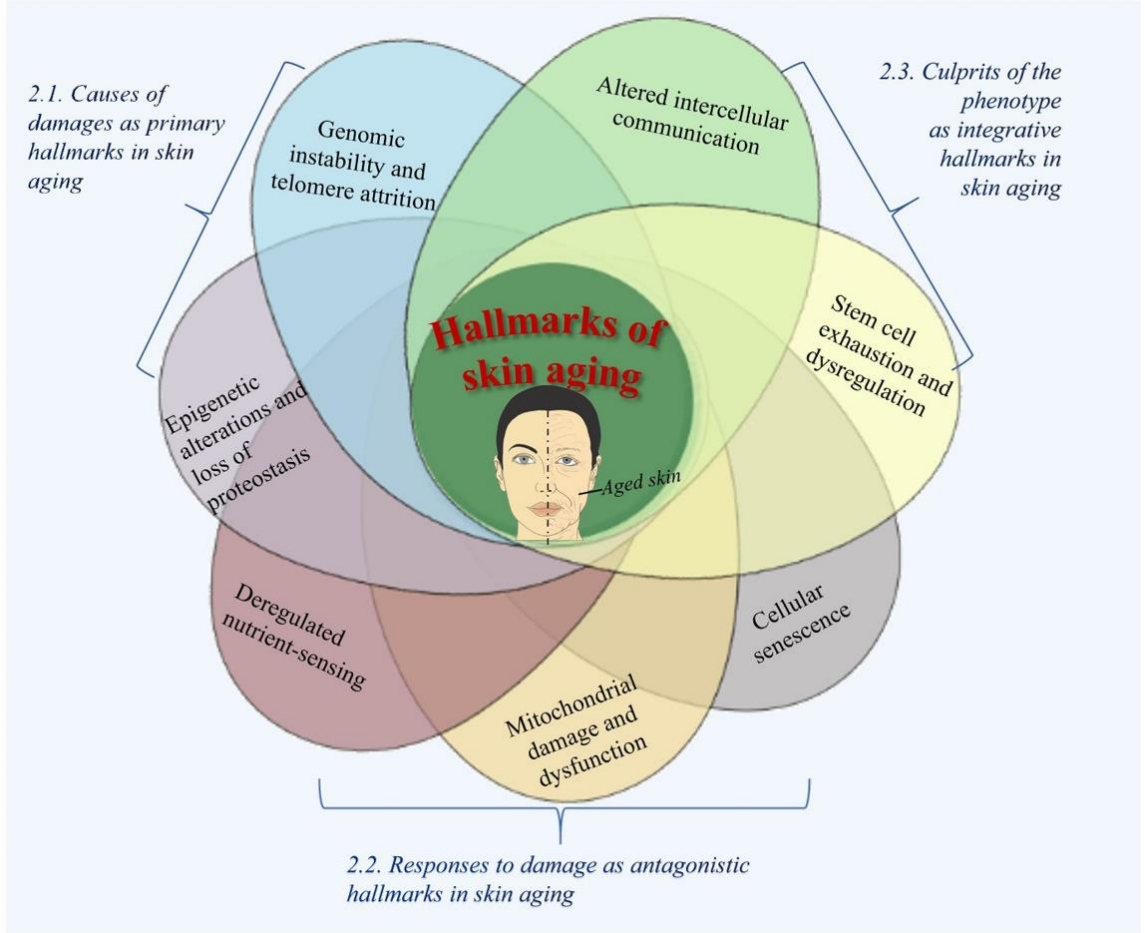
Hallmarks of aging.

Here, we attempt to propose a total of seven hallmarks that related to the process of aging in skin (Fig. 2). The seven hallmarks can generally be divided into three categories including (i) causes of damages as primary hallmarks in skin aging; (ii) responses to damage as antagonistic hallmarks in skin aging; and (iii) culprits of the phenotype as integrative hallmarks in skin aging. Each hallmark can be used alone as a hallmark of skin aging alone, but two or more hallmarks often appear at the same time due to the extensive interconnections between aging hallmarks.

## 2. Hallmarks of aging

### 2.1. Causes of damages as primary hallmarks in skin aging

#### 2.1.1. Genomic instability (DNA damage) and telomere attrition

Telomere is the repetitive DNA repeat sequences at the end of linear eukaryotic chromosomes. It is stabilised by a characteristic multi-protein complex known as the shelterin complex, preventing chromosomes from being recognized as double stranded breaks (DSBs). When a cell divides once, the telomeric repeat sequence will be lost with loss of shelterin components (that is, telomeres become uncapped), consequently the telomere will become shorter until it reaches a critical length. At this time, the cell also loses its activity and dies. Therefore, telomeres shorten is accompanied by the aging of individual cells. In other words, telomere is therefore called the "life clock" and telomere shortening is considered to be a hallmark of aging [3].

Also, telomere shortening, and its structural alterations provoke dysfunction of telomere, which may lead to replicative cellular senescence and chromosome instability in skin and is a hallmark of skin aging as well. A recent update in systemic sclerosis patients detected the shorter telomere length in blood leukocytes and estimated the role of DNA damage in human dermal fibroblasts [4]. It suggests that telomere attrition or DNA damage is one of contributors to fibroblast senescence with abnormal skin, thereby possibly providing a link between telomere shortening and skin aging as well. Telomerase is a DNA polymerase responsible for maintenance of the length of telomeres by adding telomere DNA to the end of eukaryotic chromosome to fill up the telomeres lost during DNA replication and prolong telomere repair. Telomerase deficiency or mutation is also associated with various diseases, and the role of telomerase in skin aging has been discussed in many articles [5]. In telomerase-deficient mice (*Terc*^-/-^ mice), Flores and his colleagues estimated telomere attrition and found a declined proliferative potential of epidermal stem cells [6]. In mice deficient for TRF2, TRF2 is a component of shelterin responsible for telomere capping and suppressing DNA-damage response (DDR), which is characterized by loss of skin homeostasis leading to activation of p53 signaling pathway and programmed cell death [7]. In total, accumulating evidence has shown that the loss of telomere function may lead to the decline of skin tissue function with age. Although shortened telomere plays a central role in telomere dysfunction-induced senescence, recent investigations reveal that a persistent DDR can also be activated at telomeres in cellular senescence, regardless of telomere length [8].

#### 2.1.2. Epigenetic alterations and loss of proteostasis in skin aging

Epigenetic alterations with aging are mainly based on the two regulatory mechanisms: covalent modification of histones (such as methylation, acetylation, phos-phorylation) and methylation/demethylation of cytosine residues in DNA. A recent study has showed that the DNA of the elderly has a specific hypermethylation pattern, and the methylation pattern has high tissue specificity, which indicates that DNA methylation and these changes may be caused by phenotypic changes related to skin aging [9]. With 450,000 methylation marks in various age groups from a large set of human epidermis methylomes, an interesting study defined the reduction of DNA methylation patterns and the decreased connectivity of transcriptional networks as a key feature of human skin aging [10]. Histones are defined as proteins that bind to DNA and organize chromatin. Earlier studies have documented that histone modifications comprise a vital factor in development, differentiation and maintenance of skin as evidenced by Driskell, I. et al. [11]. Histone markers, acetylated histone H4 (acH4) and histone H4 monomethylated on lysine 20 (H4K20me1), participated in Myc-induced exit from the epidermal stem cell niche and differentiate into sebocytes and interfollicular epidermis [12]. With the expression of c-Myc, mice with histone methyltransferase Ash1l mutation resulted in the imbalance of epidermal homeostasis, which is manifested as epidermal hyperplasia and delayed differentiation of keratinocytes during aging [13].

Loss of proteostasis occurs in aged organisms and cells. Progressive decline of proteasome function and altered protease secretion play established roles in the ageing process of the dermal fibroblasts leading to skin aging. Upon UV radiation, skin aging was facilitated by a decreased expression or inactivation of proteasome subunits as well as the accumulation of oxidized proteins [14]. Besides, decreased autophagy, more secreted matrix metalloproteinases (MMP) and down-regulated hyaluronic acid synthases were previously addressed in aged dermal fibroblasts, keratinocytes, melanocytes, and epidermal stem cells [15,16].

### 2.2. Responses to damage as antagonistic hallmarks in skin aging

#### 2.2.1. Deregulated nutrient-sensing in skin aging

Deregulated nutrient-sensing factors are proposed to be involved in the process of aging and skin aging. For example, insulin and IGF-1 signaling (IIS) pathway is the most common aging-controlling pathway. The dysfunction of growth hormone (GH), IGF-1 receptor, insulin receptor and up-regulation of its down-streams (such as mTOR complex), have been all related to skin aging. It has been shown that patients with primary IGF-1 deficiency showed morphological and functional changes in intrinsically aged skin, such as dryness, thinness, wrinkled skin and increased vulnerability [17]. Besides, mammalian target of rapamycin (mTOR) is an evolutionarily conserved nutrient-sensing protein kinase, which has been reported to play a key role in skin aging. A recent study has declared that mammalian target of rapamycin complex 2 (mTORC2) (one of two different enzymatic complexes of mTOR, was significantly increased in HaCaT human keratinocytes upon UVB-irradiated or aged mice skin, in which NF-κB activation and Akt/IKKα inactivation participated [18]. The role of mTOR complex in skin aging was further confirmed in an exploratory, placebo-controlled intervention trial using FDA-approved drug rapamycin [1]. Sirtuins, another nutrient-sensor in the opposite direction to IIS and mTOR, the role of which in skin aging has been well reviewed in a systemic review including 37 experimental and 16 human observation studies [19]. In skin biopsies from human donors (fetuses at pregnant age 20 to 40 week, and people from birth to 85 years old), the authors found a decreased content in sirtuin 1 accompanied by reduced proliferation of dermal fibroblasts in aged groups [20]. In parallel, another study with skin samples from females aged 20 ~ 67 also demonstrated a declined protein level of sirtuin 1 and accumulated ROS levels in elder groups [21].

#### 2.2.2. Mitochondrial damage and dysfunction in skin aging

*- Imbalanced ROS level*

Investigations to date on the relationship between mitochondrial dysfunction and skin aging has been carried out around ROS, which is a natural by-product of mitochondrial oxygen metabolism. Based on the free radical theory and the mitochondrial free radical aging theory proposed by Denham Harman in 1965 and 1972, a large amount of evidence supports the role of ROS [22], which in turn leads to mitochondrial deterioration along with overall cellular damage as a key characteristic hallmark of skin aging [23]. Then, dysfunctional mitochondria in turn promote further ROS leakage, forming a vicious circle of continuous damage accumulation. In support of this view, more recent studies have converged on the strong relationship between imbalanced ROS production and mitochondrial damage, which is a good explanation for skin aging [24].

In addition to the electron transport chain in mitochondria, ROS generation also contributes to NADP-oxidase (NOX) in the outer cell membrane. The NOX family consists of the ‘classical’ NADPH oxidase NOX1-NOX5 and the dual oxidase Duox1 and Duox2. A previous study introduced that ROS generation by NOX contributed to the expression of MMP-1 and MMP-9 induced by heat shock, which is also a hallmark of aging in keratinocyte HaCaT cells [25]. Consistent with this, a recent discovery found that inhibiting the activity of NOX can successfully save the characteristics of premature aging of skin [26]. However, the precise role of all NOX in the generation of ROS with age is still uncovered.

Besides, the integrated antioxidant defense system is also considered to be involved in prevention of skin aging. In a recent study, the activity of antioxidant defense (AD) enzymes (SOD, CAT, GSH-Px, GR, and TR) presented higher in aged rat skin [27]. It has been found that the activities of CAT and GR in the epidermis of naturally aged skin were increased and the activities of α-tocopherol, ascorbic acid and glutathion were decreased [28].

*- Mito-biogensis and mito-integrity*

Inadequete biogenesis and integrity of mitochondria can also initiate mitochondrial dysfunction and thereby result in skin aging. From the gene set enrichment analysis of the whole genome array data of human dermal fibroblasts isolated from female donors aged 20-67, a total of 137 genes significantly differed between the cells from old and young subjects. Among all the age-related transcriptome changes, the decrease of mitochondrial gene expression was the most conspicuous [21]. Mitochondrial transprition factor A (TFAM) is responsible for mtDNA replication. In mice with a keratinocyte-specific deficiency in TFAM, a higher neonatal mortality was found to be attributable to impaired epidermal barrier function [29]. In addition, primary keratinocytes originated from TFAM deficient mice failed to differentiate due to impaired oxidative phosphorylation [29], which is the bio-energetic center of the eukaryotic cell [30]. Indeed, a declined oxidative phosphorylation was also found in primary human skin fibroblasts from aged subjects [31]. At the same time, compared with young people, mitochondrial integrity of fibroblasts in the elderly was also lower [31].

*- Mitohormesis*

Mitochondrial dysfunction is also related with hormesis (hormesis in mitochondrial is called mitohormesis), which is a dose-response phenomenon that is not linear but inverted U-shaped dose response or J- or U-shaped dose response, which was firstly proposed by Southam and Ehrlich in 1943. That means mild toxic treatments may result in beneficial responses and induce a cytoprotective state, thus reducing our susceptibility to disease and possibly extending our lifespan [32]. A recent study demonstrated that L-lactate can protect skin fibroblasts against aging-associated mitochondrial dysfunction via mitohormesis, in which the intermittent shift in the intercellular redox state and the increased protective and quality control signals including the phosphorylation of AMPK were involved [33].

#### 2.2.3. Cellular senescence in skin aging

Cellular senescence is also a hallmark of aging, which was formally described by Hayflick in 1961 in normal human fibroblasts with a finite proliferative capacity. Later, it was demonstrated by Bodnar in 1998 that telomere shortening is a molecular clock that accelerates senescence. In addition to telomere shortening, cellular senescence can also be caused by DNA damage, cell cycle arrest (p21, p53 markers) and many other stressors [34]. A collection of markers such as p16^INK4A^, HMGB-1, senescence-associated β-galactosidase (SAβGAL) and lamin B1 have been accepted to define senescence, that is, the expression or suppression of these markers indicates that cells are undergoing senescence. p16^INK4A^, a senescence biomarker, its expression directly correlates with chronological aging of human skin [35]. Recently, it has been widely demonstrated that the accumulation of melanocytes expressing p16^INK4a^ in human skin epidermis is significantly correlated with the increase of facial wrinkles, the higher age perception and the age-related elastin morphology in the dermal papilla [36]. Additionally, histochemical detection of SAβGAL is the most used assay for senescence, which is also frequently applied in human dermal fibroblasts [37]. The nuclear matrix protein Lamin B1 is another marker to quantify senescence in skin, and the decreased level of Lamin B1 induced by UVB radiation in keratinocytes indicated cellular senescence and skin aging [38]. Besides, there are several novel findings on senescence markers for aging. A recent study demonstrates that the p53 dependent gene Ras-related associated with diabetes (RARD) in senescent skin cells from aged human donors is a novel senescence, and its deletion can exaggerate H_2_O_2_-induced senescence [39].

It is worth mentioning that senescent cells accumulate in the aged skin and express an altered secretome, which is called senescence-associated secretory phenotype (SASP). SASP can be cytokines, chemokines, growth factors or proteases that secreted upon cell senescence driven by diverse DNA damage stimuli, including ultraviolet radiation, telomere attrition caused by normal aging, activated oncogenes and cancer treatment. In murine demal fibroblasts, it has been proved that DDR increases SASP with elevated IL-6 and IL-8 through NF-κB signaling pathway and also provokes chronic inflammation [40]. Additionally, in aged melanocyte with increased expression of p16^INK4A^ and reduced expression of HMGB1, SASP caused paracrine telomere dysfunction and limited the proliferation of bystander cells via increased mitochondrial ROS in CXCR3 activation manner [41]. However, a different finding is that DDR-stimulated cell senescence is accompanied by the high expression of senescence marker p16^INK4a^ in primary human fibroblasts and mammary epithelial cells, but it is independent of SASP [42], indicating that the involvement of SASP in cellular senescence is highly dependent on the cell type and the senescence-induced stimulus.

### 2.3. Culprits of the phenotype as integrative hallmarks in skin aging

#### 2.3.1. Stem cell exhaustion/dysregulation

Since the discovery of pluripotent stem cells by Till and McCulloch in 1961, stem cells has been known to play a central role in replacing cells in tissues and their competition coordinates homeostasis and ageing. That is, exhaustion or dysregulation of stem cells is a common culprit of aging and also a comprehensive consequence of a variety of aging-related injuries. Just as alveolar stem cell exhaustion can eventually lead to pulmonary diseases, skin stem cell exhaustion can also lead to a variety of skin disorders and skin aging, manifested as skin atrophy, fragility, dyspigmentation and delayed wound healing. Epidermal stem cells are the main stem cells in the skin. In vivo analysis of keratinocyte stem cell of the murin hair gollicle revealed that they exhibit permanent DDR, leading to the hemi-desmosomal structural protein collagen 17A1 (COL17A1) proteolysis, which is a key molecule for hair gollicle stem cell (HFSC) maintenance in triggering HFSC aging [43]. And recent evidence on epidermal stem cells and skin aging found that loss of COL17A1 may cause junctional epidermolysis bullosa, and exhaust adjacent melanocytes and fibroblasts with a reduced number and a declined regenerative capacity of epidermal stem cells, thereby resulting in skin aging manifested as atrophy [44]. Indeed, the abnormal behavior of epidermal stem cells can be attributed to altered telomerase activity and shortened telomere length, DNA repair defects and oxidative stress [6, 44]. A deeper insight on the molecular mechanism revealed that Jak-Sat signaling participated in and promoted a feedback loop in aged Krt-15-GFP-positive stem cells (one of the best-charaterized stem cell in skin) in response to pro-inflammatory environment with increased levels of protein cytokines (such as BLC/CXCL13, GM-CSF, ICAM-1/CD54, IL-1α), proving that age-related inflammation may inhibit the function of epidermal stem cells [45]. Also, autophagic response is involved in DNA mutant expressed in skin epidermal stem cells with declined mitochondrial activity, over-produced ROS and increased cell death. Most notably, due to the vital role of stem cells in skin, recent advances in the application of various stem cells in skin regeneration and rejuvenation against skin aging is increasing.

**Table 1:** Summary of the *in vivo* studies on each hallmark of skin aging.

Hallmarks	Test models	Key observations	Ref
*Genomic instability (DNA damage) and telomere attrition*			
Shorter telomere length	Blood leukocytes from SScs patients *vs.* healthy controls	Oxidative DNA damage leading to skin fibrosis ↑ in SSc	[4]
Telomere attrition	*Terc*^-/-^ mice	Proliferative potential of epidermal stem cells ↓	[6]
DDR	*TRF2^∆/∆^ K5-Cre* mice	Partial embryonic lethality↑; skin embryonic development↓	[7]
*Epigenetic alterations and loss of proteostasis*			
A specific hyper- methylation pattern	Suction blisters from the volar forearms of healthy male volunteers	Age-related hypermethylation in human epidermis samples analyzed by platform for genome-scale	[9]
Loss of epigenetic regulatory fidelity	Epidermal samples from healthy volunteers (18-79 years)	In elder groups: DNA methylation patterning↓ and connectivity of transcriptional networks ↓	[10]
Histone modifications	Dorsal and tail skin from K14MycER transgenic mice and WT mice	Myc-induced exit from the epidermal stem cell niche and differentiate into sebocytes and interfollicular epidermis	[12]
	Mice with histone methyltransferase Ash1l mutation	The imbalance of epidermal homeostasis	[13]
Loss of proteostasis	Skin tissues from healthy male volunteers (9-18 years *vs* 50-94 years)	In elder groups: autophagy related; proteasome subunits↓	[15]
*Deregulated nutrient-sensing in skin aging*			
Increased mTORC2 pathway	C57BL/6 mice (12 and 24 months, male)	mTORC2 expression and activity and the levels of IKKα and p65 phosphorylation in aged skin ↑; by UVB irradiation in HaCaT cells: the mTORC2/Akt/IKK signaling pathway↑ and NF-κB activated	[18]
Lower level of SIRT 1	Skin biopsies of human (fetuses at pregnant age 20-to-40-week, people from birth to 85 years old)	Age-related: sirtuin 1 content ↓; proliferation of dermal fibroblasts ↓	[20]
	Skin biopsies of females aged 20-67	Age-related: sirtuin 1 protein level ↓; ROS level ↑	[21]
*Mitochondrial damage and dysfunction in skin aging*			
Oxidative stress in mitochondria	Skin from hairless mice expressing roGFP1 vs WT mice exposed to UVA or blue light	In blue light-exposed groups: mitochondrial oxidative stress ↑; oxidizes keratinocytes ↑; ROS production ↑	[23]
	human skin exposed blue light	Oxidizes keratinocytes; ROS production ↑; flavin autofluorescence ↓	
mtDNA common deletion	Normal adult volunteers (20 ~30-year-old *vs* ≥ 80-year-old)	In aged and sun-exposed subjects: mtDNA common deletion ↑; Lat-A treated cells have a low relative cell surface area but a high mtDNA common deletion level; ROS/oxidative stress ↑ → mtDNA common deletion ↑	[24]
Imbalanced ROS level	Skin biopsies from *Xpc^-/-^*mice (4-month-old *vs* 1.5-year-old)	Steady-state ROS levels ↑; aging markers ↑ (p16INK4a expression and SA-b-gal activity) and NOX activity ↑ in aged Xpc^-/-^mice groups	[26]
Increased activity of antioxidant defense enzymes	Skin from healthy Wistar rats (from 3 days to 21 months, male)	Skin aging markers in aged rats: the epidermal layer thickness and the intercellular space throughout the epidermis ↑; in epithelial-dermal junction ↓; intracytoplasmic vacuolization ↑; Oxidative stress markers in aged rats: the expression of PCNA ↓; 8-OxoG expression↑; SOD, CAT, TR activities ↓ and GSH-Px and GR activities ↑	[27]
Decreased expression of mitochondrial genes	Human (20-67 years-old, female)	In aged groups: ROS-production; NAD^+^-dependent SIRT 1 ↓; MUSK, GPIK5, SETDB2, PCSK6 and STRA8 gene expression ↑; TAF6, AKF1S1, AK1RIN 2, DRD5 and HNF4A gene expression ↓	[21]
Decreased mitochondria-derived ROS	WT *vs TFAM* deficient mice	In *TFAM* deletion mice: cellular metabolism ↓ (oxygen consumption and superoxide ↓) and ROS generation ↓; hair follicles growth and development↓; epidermal differentiation ↓	[29]
Declined mitochondrial integrity	Primary dermal fibroblasts from human donors (17-84 years of age, male; 20-70 years of age, female)	In aged groups: mitochondrial ETC subunits gene expression ↓; oxidative phosphorylation and mitochondrial efficiency (oxygen consumption) ↓	[31]
*Cellular senescence in skin aging*			
Higher levels of p16INK4a positive cells	Middle-aged offspring with their partners (varied from 46 to 81 years of age, average of 63 years)	In elder groups: p16INK4a positive cells ↑	[35]
Decreased level of Lamin B1	participants (men and women, aged 45-81 years, has a long-lived family or consisted of their partners)	In aged groups: dermal p16INK4a ↑; altered elastic fiber morphology ↑	[36]
	Mice	mRNA of PAI-1 (Serpine1); p16 and p19 (Cdkn2A) ↑; Lamin A/C protein levels ↓	[38]
*Stem cell exhaustion/dysregulation*			
Hair follicle stem cell (HFSC) aging	Col17a1 cKO *vs* C57BL/6 wild-type mice	In elder groups: fluorescence intensity of COL17A1 ↓; In Col17a1 cKO mice: hair loss and grayin ↑, c-MYC expression ↑; COL17A1 proteolysis in HFSCs triggers HFSC aging; DNA damage ↑ and sustained DDR accumulated ↑	[43]
	Scalps from human donors (22- 70 years old, female)	In elder groups: miniaturized HFs ↑; COL17A1, K15, and CD200 expression ↓; the size of the K15+ bulge region ↓; the number of DNA damage foci in bulge keratinocytes ↑	
Stem cell delamination	Tail skin from WT (young *v*s aged mice); Tail skin from WT *vs* Col17a1 cKO mice; Tail skin from WT *vs* hCOL17A1 tg mice	In aged WT mice: the epidermal thickness in tail scale areas ↑; the numbers of hemidesmosomes ↑ and micro-delaminations at the basement membrane in tail scale basal cells ↓; In hCOL17A1 tg mice: the epidermal thickness in the tail scale area ↑; the number of PDGFRa^+^ dermal fibroblasts, number of KIT^+^; epidermal melanocytes ↑	[44]
Declined function of stem cells	Skin from WT *vs* aged Krt-15-GFP mice	The number of Krt-15-GFP hair follicle stem cell ↑; Age-associated functional (by colony assays) in Krt-15-GFP stem cells ↓;	[45]
*Altered intracellular communication*			
Aberrant collagen homeostasis in dermal fibroblasts	Healthy adult human volunteers	Type III procollagen in human skin dermal fibroblasts ↓; MMP in human skin dermal fibroblasts ↑; IL-1β and IL-6 in human skin dermal fibroblasts ↑	[47]
Reduced generation of long-lived memory T-cells	Healthy adults (<35 years old *vs* >60 years old)	In aged old groups: dysfunctional CD4^+^CD28^-^ T cells ↑; CD8^+^ among CD3^+^ T-cells ↓; naive T-cells ↓; inhibitory PD1 and the ecto-nucleotidase CD39 marker expression ↑; skin ILC2 ↓	[48]

#### 2.3.2. Altered intercellular communication

Intercellular communication is a signal sent by one cell and transmitted to another cell through kinds of media, such as chemical signal molecules, adhesion of molecules on the surface of adjacent cells and adhesion between cells and extracellular matrix. During aging, with the inflammatory reactions, dysregulation of signaling molecules (e.g. insulin-IGF1 signaling, renin-angiotensin, EGF-signaling) via endocrine or paracrine may affect the microenvironment of surrounding tissues, and finally result in the disorganization and dysfunction of all tissues [3]. Inflammaging is a concept previously proposed by Franceschi in 2000, characterized by chronic and age-related low-level inflammation and, its alterations are related to intercellular communication in recent updates. It is commonly due to the accumulation of pro-inflammatory tissue damage and circulating cytokines or the decline of immune system function, and may cause a series of various diseases, such as diabetes, cardiovascular disease and skin disorders as well [46]. Recent evidence for an inflammaging phenotye in aged skin demonstrated the central role of senescence in skin inflammaging. For example, fibroblasts from aged skin expressed various SASP (such as MMP, IL-1β, IL-6), which is a general senescent marker, will affect nearby microenvironment, eventually led to extracellular matrix remodeling and exacerbated skin aging [47]. A recent literature around the central role of immune cells in skin inflammaging revealed that skin samples from aged subjects depicted dysfunctional CD4+ and CD8+ T cells and an upregulation of immunosuppressive receptor PD-1 that suppress the adaptive immune response [48], that may also contribute to skin inflammaging via releasing a pro-inflammatory T helper (Th)17 phenotype [49]. Besides, extracellular vesicles (EVs) are suggested to be prominent messengers and new members of SASP, which transmit information through their miRNA cargo between cells and drive intercellular communication. It has been found that small EVs-packaged miRNAs are transferred from senescent fibroblasts to keratinocytes and also influence keratinocyte behavior *in vitro* [50], that shed a light on the EVs and their cargos are contributors to human skin homeostasis during aging.

## 3. Summary and perspectives

Here, we summarized three categories and seven hallmarks of skin aging (Fig. 2). Although each hallmark can be used as an index to characterize skin aging (Table 1), it cannot comprehensively explain all the phenomena of aging in skin. Even from each hallmark we reported separately above, it is also obviously shown that there is no doubt that none of each hallmark is performed alone. In most cases, it is a combined performance of several hallmarks of skin aging during the process of skin aging. For example, air pollution (e.g., particular matter, ozone, NO_2_ and CO_2_) is known to be one contributor to skin aging. A recent report from two German cohort studies has reported that tropospheric ozone can aggravate wrinkle formation [51], which is possibly caused by increased oxidative stress that is accompanied by depleted antioxidants and increased expression of MMPs (markers of cellular senescense) [52-54]. A more recent review has further well summarized an integrated occurrence of several hallmarks in keratinocytes, fibroblasts and melanocytes senescence induced by air pollution, including inflammation, protein quality control, mitochondrial dysfunction, ROS, and SASP [55]. Additionally, an earlier study revealed that aged dermal fibroblasts can promote age-associated secretory phenotyes (e.g. reduced collagen, increased MMPs and increased cytokines), and finally result in aging of skin connective tissue. It indicates that the hallmarks of cellular senescence have participated in intercellular communication, therefore leading to thin and damaged dermis [47].

Up to date, most studies have been limited to ‘verifying’ the hallmarks of skin aging and have tried their best to organize the relationships between upstream and downstream, rather than exploring the ‘new findings’ of skin aging hallmarks. It should be recognized that the hallmarks of skin aging should not be restricted to these seven hallmarks. Therefore, more research on cellular and molecular mechanism is required to be insistently continued to develop the investigation on the hallmarks of skin aging.
